# An Immunomodulatory Polysaccharide–Protein Complex Isolated from the Polypore Fungus *Royoporus badius*

**DOI:** 10.3390/jof9010087

**Published:** 2023-01-06

**Authors:** Bryan C. C. Lim, Mehreen Zeb, Wai-Ming Li, John Z. Tang, Christian Heiss, Linda E. Tackaberry, Hugues B. Massicotte, Keith N. Egger, Kerry Reimer, Parastoo Azadi, Chow H. Lee

**Affiliations:** 1 Department of Chemistry and Biochemistry, Faculty of Science and Engineering, University of Northern British Columbia, Prince George, BC V2N 4Z9, Canada; 2 Complex Carbohydrate Research Centre, University of Georgia, Athens, GA 30602, USA; 3 Department of Ecosystem Science and Management, Faculty of Environment, University of Northern British Columbia, Prince George, BC V2N 4Z9, Canada

**Keywords:** *Royoporus badius*, *Picipes badius*, *Polyporus badius*, polysaccharide–protein, immunomodulation

## Abstract

Many wild edible polypore mushrooms have medicinal value. In this study, we investigate the potential medicinal properties of the wild polypore mushroom *Royoporus badius* collected from north-central British Columbia, Canada. Water extract from *R. badius* was found to exhibit potent immunomodulatory activity. The extract was purified using DEAE-Sephadex anion-exchange chromatography as well as Sephacryl S-500 and HPLC BioSEC5 size-exclusion chromatography, to yield a novel polysaccharide-protein complex (IMPP-Rb).IMPP-Rb has a peak maxima molecular weight (M_p_) of 950 kDa. GC-MS analyses showed that IMPP-Rb is composed predominantly of glucose (49.2%), galactose (11.3%), mannose (10.8%), rhamnose (9.6%), and galacturonic acid (8.2%), with smaller amounts of xylose (5.2%), fucose (2.8%), N-acetyl glucosamine (1.8%), and arabinose (1.2%). IMPP-Rb has multiple linkages, with 4-Glc*p*, 4-Man*p*, 6-Man*p*, 3,4-Man*p*, 4-Xyl*p*, and 2-Rha*p* being the most prominent. IMPP-Rb is capable of inducing many cytokines in vitro and the protein component is indispensable for its immunomodulatory activity. IMPP-Rb has potential application as an immuno-stimulatory agent with pharmaceutical value.

## 1. Introduction

Fungi continue to represent a largely untapped resource for drug discovery. This is because only 10% of the approximately 150,000 macrofungi have been taxonomically described [1]. Therefore, more extensive collection, identification, and investigation of mushrooms is strongly encouraged. Recently, we took the initiative to explore wild mushrooms native to British Columbia (BC), Canada for medicinal properties [2,3,4]. We documented that the antiproliferative and immunomodulatory activities of 20 out of 29 (69%) species of mushrooms from north-central BC and Haida Gwaii had not been reported [2,3].

Polypores are a group of fungi that form tough or leathery fruiting bodies with tubes and pores on the underside. Most polypores are considered non-toxic and some are categorized as medicinal mushrooms, with notable examples including *Ganoderma lucidum* and *Trametes versicolor* [5]. The fungus selected for the present study was *Royoporus badius* A.B. De. *R. badius*, commonly known as the black-footed polypore, in the family Polyporaceae, which is found in the temperate areas of Asia, Australia, Europe, and North America [6]. The black-footed polypore has a complex taxonomic history, and can be found in the literature under different names. In addition to *R. badius*, it has been known as *Polyporus badius* (Pers.) Schwein., and most recently as *Picipes badius* (Pers.) Zmitr. & Kovalenko. However, as the latter name is not yet widely accepted as valid, we will use the name *Royoporus badius* for the remainder of this article.

The major bioactive components in medicinal and edible fungi and mushrooms are polysaccharides and polysaccharide–protein complexes. These macromolecules have attracted attention due to their diverse bio-pharmacological activities, high potency, and non-toxicity [7,8,9]. The most widely studied polysaccharides or polysaccharide–protein complexes are derived from *Trametes versicolor*, *Lentinula edodes*, *Ganoderma lucidum*, *Phellinus* spp., and *Agaricus* spp., and their documented biological activities include immunomodulatory, anti-cancer, anti-inflammatory, anti-oxidant, hypoglycemic, hepatoprotective, and neuroprotective effects [7,8,9,10,11]. To date, studies have suggested that the immunomodulating function of mushroom polysaccharides or polysaccharide–protein complexes is associated with their effects on both the innate and adaptive immunity systems, and this is related to their in vivo anticancer activity. For instance, polysaccharide-K (PS-K) from *T. versicolor* is a polysaccharide–protein complex containing a D-glucose backbone that can induce expression of several cytokines such as TNF-α, IL-1, IL-8, and IL-6 in various immune cells, including macrophages, in vitro or in vivo [7]. It is generally believed that these cytokines assist in increasing the capacity of the host immune system to target tumors by directly stimulating cytotoxic T-cells, enhancing antibody production in B lymphocytes and IL-2 receptor expression in T-lymphocytes [7]. Three mushroom polysaccharides or polysaccharide–protein complexes (including PS-K) have shown effectiveness in humans and are currently in use as adjuvants for chemotherapy in China and Japan [7,8,12].

Macrophages are regarded as a first line of defense against foreign pathogens and are the major immune cells involved in innate immunity, acting by engulfing microbes and secreting proinflammatory factors such as TNF-α [13]. Macrophages can also act as antigen-presenting cells to activate T cells and initiate adaptive immune responses, thereby serving to bridge innate and adaptive immunity [13]. The murine macrophage cell line RAW264.7 has been widely used as a model system to study in vitro the mechanisms of macrophage activation in immuno-regulation [14], and the induction of TNF-α production as an indicator of immunomodulation [15]. In this study and in our previous research [16], increased TNF-α production and secretion into the media by RAW264.7 cells was used as an indicator of immunomodulatory activity. Given that TNF-α is a proinflammatory factor, it has also been used as an inflammatory marker [17], especially in screening natural polysaccharides for their ability to inhibit lipopolysaccharide-induced TNF-α production as an indicator of anti-inflammatory activity [3,17,18].

Limited documentation exists to describe the potential medicinal properties of *R. badius*; its ethanol extract has been shown to possess antibacterial activity, while its water extract demonstrated anti-oxidant activity [19,20,21]. Given the limited studies, especially concerning the isolation of potential medicinal compounds, we hypothesized that *R. badius* contains compound(s) with anti-cancer properties. Therefore, we collected the polypore from north-central BC and screened its extracts for three major bioactivities related to cancer treatment, including immunomodulatory activity. In this study, we found that the water extract of *R. badius* has potent immunomodulatory activity. Using DEAE-Sephadex, Sephacryl S-500, and HPLC BioSEC5 chromatography, we isolated an immunomodulatory polysaccharide–protein complex from *R. badius*. Structural analyses were performed, including gas chromatography–mass spectrometry (GC-MS) to determine its monosaccharide content and glycosidic linkages, and Fourier transform infrared (FTIR) spectroscopy to identify its functional groups.

## 2. Materials and Methods

### 2.1. Material and Chemicals

Dextran standards (T1, T5, T12, T25, T50, T80, T150, T270, T410) were purchased from Sigma-Aldrich (St. Louis, MO, USA). Fetal bovine serum was from Life Technologies Inc. (Waltham, Massachusetts, USA) and Dulbecco Modified Eagle Medium from LONZA (Walkersville, Maryland, USA). The DEAE-Sephadex and HiPrep 26/60 Sephacryl™ S-500 HR were from GE Healthcare (Uppsala, Sweden). Polysaccharide-K was purchased from Kureha Pharmaceuticals (Japan). The HPLC BioSEC-5 column and its guard column were purchased from Agilent (Santa Clara, CA, USA).

### 2.2. Identification of Mushroom Species

The collected mushrooms were initially identified as *Royoporus badius*, based on morphological characteristics. Specimens CL86, CL150, and CL152 were collected in August 2016, June 2017, and July 2018, respectively, from Wilkins Regional Park, Miworth, BC. The voucher specimens CL86, CL150, and CL152 were deposited at the University of Northern British Columbia, Canada. All collections were confirmed by DNA sequencing. Genomic DNA extraction and polymerase chain reaction (PCR) amplification were conducted as described previously [2,3]. DNA sequences of the internal transcribed spacer 2 (ITS2) of the 28S gene were aligned and edited using CLC Main Workbench (Qiagen, Carlsbad, CA, USA). The DNA sequences of collections CL86, CL150, and CL152 were identical and had high sequence similarity to *R. badius* GenBank KM411465.1 (98% identity/87% coverage).

### 2.3. Preparation of Crude Extracts from R. badius

The fungal specimens were dried and then crushed into powder, then sequentially processed into four extracts as previously described [2,3]. For large extraction, 350 g of powdered specimen was processed with 3.5 L of 80% ethanol for 3 h at 65 °C. The solution, referred to as 1A, was filtered through Whatman paper No. 3. The residue was subjected to a second step of 50% methanol extraction for 3 h at 65 °C. This filtrate was designated as 1B. The residue was subjected to a third step which involved water extraction for 6 h at 65 °C. The filtrate from the water extraction was referred to as 1C. The residue was subjected to the final step of 5% sodium hydroxide (NaOH) extraction at 65 °C for 6 h. The filtered solution from this step was referred to as 1D. All extracts were subjected to roto-evaporation followed by lyophilization. All lyophilized extracts were reconstituted in water at 20 mg/mL and were filter-sterilized using a 0.2 μm filter (Sarstedt, Quebec) before they were assessed for immunomodulatory and antiproliferative activity as described below.

### 2.4. Cell Line and Assessment for Immunomodulatory Activity

RAW264.7 mouse macrophage cell line was purchased from the American Type Culture Collection (Rockville, MD, USA) and maintained in Dulbecco Modified Eagle Medium. Cells were grown in medium supplemented with 10% fetal bovine serum in a humidified incubator at 37 °C supplied with 5% carbon dioxide. To assess immunomodulatory activity, we measured TNF-α production in RAW264.7 cells upon treatment for 6 h with 1 mg/mL fungal extracts or semi-purified or purified IMPP-RB. Fifty µL of medium was removed and stored at −80 °C until TNF-α content determination using enzyme-linked immunosorbent assay (ELISA) was performed as previously described [2,3]. To measure other cytokines and chemokines, a mouse cytokine/chemokine 32-plex array was conducted at Eve Technologies (Calgary, AB, Canada).

### 2.5. Cell Line and Assessment for Antiproliferative Activity

The effects of crude extracts on the viability of HeLa human cervical cancer cells was determined by 3-(4,5-dimethylthaizaol-2-yl)-2,5-diphenyl tetrazolium bromide (MTT) assay as described previously [2,3,22]. Cells were plated at a density of 1.5 × 10^3^ cells/well in 100 µL media in 96-well plates and treated after 24 h with various concentrations of filter-sterilized extracts. After a further 48 h, cells were subjected to MTT assay. For time-dependent experiments, cells were plated at 1.0 × 10^3^ cells/well, and crude extracts were added 24 h later. The MTT assay was performed on Day 0, taken as the day on which the extracts were added, and again daily for up to 7 days.

### 2.6. Purification of Immunomodulatory Polysaccharide

The crude water extract 1C was loaded at about 0.86–1.0 g onto the DEAE Sephadex, which was designated column 2 (750 mL bed volume), using the XK-50 column (50 mm × 100 cm) equilibrated with 20 mM bis-tris buffer pH 6.5, at a flow rate of 1.0 mL/min. After washing with the same buffer, column 2 was eluted with two bed volumes (2 L) containing 1 M NaCl. The collected eluent was lyophilized, dialyzed, re-lyophilized, and reconstituted in 2 mL 150 mM NaCl at a concentration of 50 mg/mL before loading onto a pre-packed Sephacryl S-500 (1.0 × 30 cm), which was designated column-3, at a flow rate of 1.3 mL/min using an AKTA Pure system (GE Healthcare). The fractions collected from column 3 were subjected to measurement of carbohydrate and protein content in addition to assessment for the ability to induce TNF-α in RAW264.7 cells. Carbohydrate and protein contents in the fractions were determined using the phenol–sulfuric acid method [23] and a Pierce BCA protein assay kit (Waltham, MA, USA), respectively. Fractions containing immunomodulatory activity were pooled, lyophilized, and were designated IMPP-Rb, then resuspended in water and subjected to HPLC BioSEC5, and designated column 4, as described below.

### 2.7. Molecular Weight Distribution and Purification by HPLC

HPLC BioSEC5, referred to here as column 4, was used for the following purposes: (i) to further purify the bioactive compound, (ii) to estimate the purity of the sample, and (iii) to determine the molecular weight of the sample. Column 4 (5 µm, 500 A, 4.6 × 300 mm), equipped with a guard column (5 µm, 500 A, 4.6 × 50 mm), was operated using an Agilent HPLC 1200 series system. Refractive index detector (Agilent 1200 Infinity II series, G7162A RID) with a standard optical unit was used for detection purposes. The temperature was maintained at 28 ± 0.2 °C. The peak maxima molecular weight (M_p_) was estimated by calibration with T-series Dextran standards (150–2000 kDa). Column 4 was equilibrated with mobile phase to obtain a stable baseline. Ten µL of 10 mg/mL IMPP-Rb was injected through an autosampler (Agilent 1200 series G1329A) and water was used as the mobile phase (flow rate = 0.4 mL/min). After the HPLC profile was obtained, the respective peaks were purified by fraction collection. Fraction-collected peaks were re-injected to confirm sample purity. The peaks were assessed for immuno-stimulatory activity, as described below. Fractions containing bioactive peaks were lyophilized and subjected to monosaccharide composition and linkage analyses, as described below.

### 2.8. Monosaccharide Composition Analysis

Monosaccharide content of HPLC-purified samples was determined by GC-MS, as previously described [16,24]. IMPP-Rb (300 µg) and internal standard inositol (20 µg) were subjected to acid methanolysis by heating with 1 M methanolic HCl in a sealed screw-capped glass tube (18 h, 80 °C). The samples were dissolved under a nitrogen stream and treated with methanol, pyridine, and acetic anhydride for 30 min. Excess solvent was removed and samples were subjected to derivatization with Tri-Sil HTP (80 °C, 30 min). The resulting per-O-trimethylsilyl (TMS) derivatives of the monosaccharide methyl glycosides were analyzed using GC-MS (Agilent 7890A GC, 5975C MSD) on a Supelco Equity-1 fused silica column (30 m × 25 mm ID).

### 2.9. Methylation and Linkage Analysis

Glycosyl linkage analysis was conducted using GC-MS, as previously described [12,13]. This was achieved by methylating 0.98 mg of IMPP-Rb with dimsyl potassium (15 min) and methyl iodide (15 min) in a screw-capped glass tube. After extraction with dichloromethane and reducing the methyl carboxylates with LiAlD_4_ in THF for 4 h at 100 °C, the sample was permethylated with NaOH (15 min) and methyl iodide (45 min), depolymerized with 400 µL of 2 M TFA in a sealed tube (2 h, 121 °C), reduced with NaBD_4_, and acetylated with a mixture of acetic anhydride and TFA (25 min, 50 °C), resulting in partially methylated alditol acetates (PMAAs). The sample was dried under N_2_, reconstituted in dichloromethane (DCM), and washed with nanopure water. The resulting DCM solution was subjected to GC-MS analysis using a 30 m Supelco SP-2331 fused silica column on an Agilent 7890A GC-MS instrument (Agilent 5975C MSD with an electron-impact ionization source).

### 2.10. FTIR Spectroscopy Analysis

FTIR spectroscopy was conducted to analyze the functional groups, using a Bruker ATR-FTIR spectrophotometer (Billerica, MA, USA) with a detection frequency from 4000−400 cm^−1^. For FTIR, a small amount of each sample was placed on a diamond window and analyzed using OPUS software. Twenty-two scans were performed in order to obtain a representative FTIR spectrum.

### 2.11. Proteinase-K Enzyme Assay

IMPP-Rb (1.5 mg/mL) and polysaccharide-K (0.2 mg/mL) were treated with proteinase-K (1 mg/mL) for 2 h at 37 °C in a final volume of 350 µL. The sample–enzyme mixture was then subjected to heat deactivation at 80 °C for 20 min. The mixtures were stored at 4 °C prior to addition (100 µL) to RAW 264.7 cells for assessment of immunomodulatory activity, as described above.

## 3. Results

### 3.1. Identification of Royoporus badius

Based on morphological characteristics, the collected fungi were tentatively identified as *Royoporus badius*. This was further confirmed by DNA sequencing analysis of the ITS2 region and the 5′ end of the D1-D2 region of the 28S gene, using the ITS3 and NLB4 primers, followed by a BLAST search of GenBank. The DNA sequences of collections CL86, CL150, and CL152 were identical and had high sequence similarity with collections of *R. badius* or one of its synonyms.

### 3.2. Assessment of Crude Extracts for Immunomodulatory and Antiproliferative Activities

**Figure 1:**
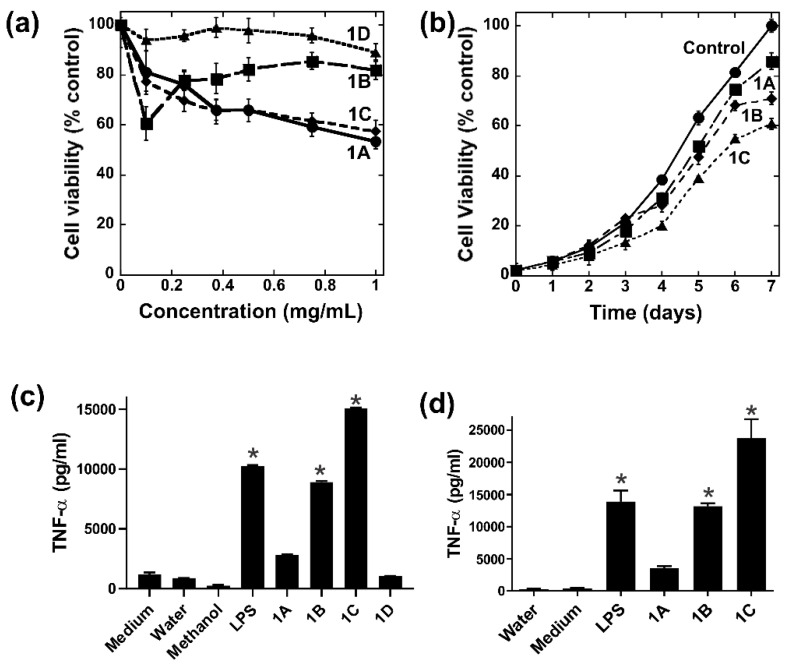
Immunomodulatory and antiproliferative activities of crude extracts obtained from *R. badius*. (**a**) Dose- and (**b**) time-dependent MTT cell viability assays on HeLa cells. The concentration used for the time-dependent experiment was (**b**) 0.5 mg/mL. At 1 mg/mL, crude extracts from specimens (**c**) CL86 and (**d**) CL152 were assessed for their ability to induce TNF-α production in RAW264.7 macrophage cells, as an indicator of immunomodulation. Lipopolysaccharide (LPS) was used as a positive control, while media, water, and methanol were used as negative controls. Results presented are representations of two separate experiments (*n* = 2). Error bars show SD. One-way ANOVA (Tukey test) was used for statistical analysis. * *p* < 0.05 compared with the water control.

Powdered *R. badius* was sequentially extracted with 80% ethanol (1A), 50% methanol (1B), water (1C), and 5% NaOH (1D), then assessed for antiproliferative and immunomodulatory activities as shown in Figure 1. Dose- (Figure 1a) and time-dependent (Figure 1b) MTT assays showed that 1A, 1B, and 1C from *R. badius* only had modest antiproliferative effects on HeLa cells, while 1D exhibited no activity (Figure 1a). In contrast, 1B and 1C from *R. badius* showed potent immunomodulatory activity in specimens CL86 (Figure 1c) and CL152 (Figure 1d); 1C in particular had stronger activity than the positive control lipopolysaccharide. The ethanol extract 1A from both specimens had modest immunomodulatory activity (Figure 1c,d).

### 3.3. Purification of Immunomodulatory Polysaccharide IMPP-Rb from R. badius

**Figure 2:**
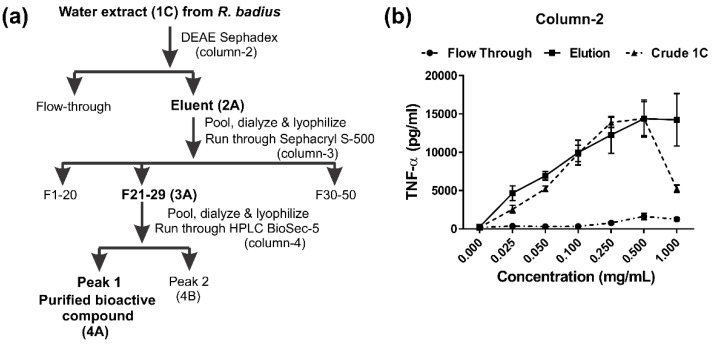
Purification of IMPP-Rb from *R. badius*. (**a**) Summary of the purification scheme used. (**b**) Purification using DEAE-Sephadex (column 2). Flow-through and eluent (post- 1 M NaCl) from a 750 mL DEAE column were pooled, dialyzed, and lyophilized. Various concentrations of samples were assessed for immunomodulatory activity, as indicated. Results shown are representative of two separate experiments.

The water extract 1C had the strongest immunomodulatory activity, and was therefore selected for further purification of the relevant compounds. The overall scheme adopted to purify the immunomodulatory compound from *R. badius* is illustrated in Figure 2a. The extract 1C was subjected to the first purification step using column 2 (DEAE-Sephadex anion-exchange chromatography). Multiple buffers including piperazine (pH 5.3), bis-tris (pH 6.5), triethanolamine (pH 7.7), propane-1,3-diamino (pH 8.9) and ethanolamine (pH 9.5) were first assessed to determine the optimum buffer for use in purification. When using bis-tris, triethanolamine, or ethanolamine, bound bioactive compounds were eluted with 1 M NaCl and exhibited the strongest immunomodulatory activity. Bis-tris was selected as a buffer of choice because it took less time to equilibrate the column, due to its lower pH (pKa) value.

**Figure 3:**
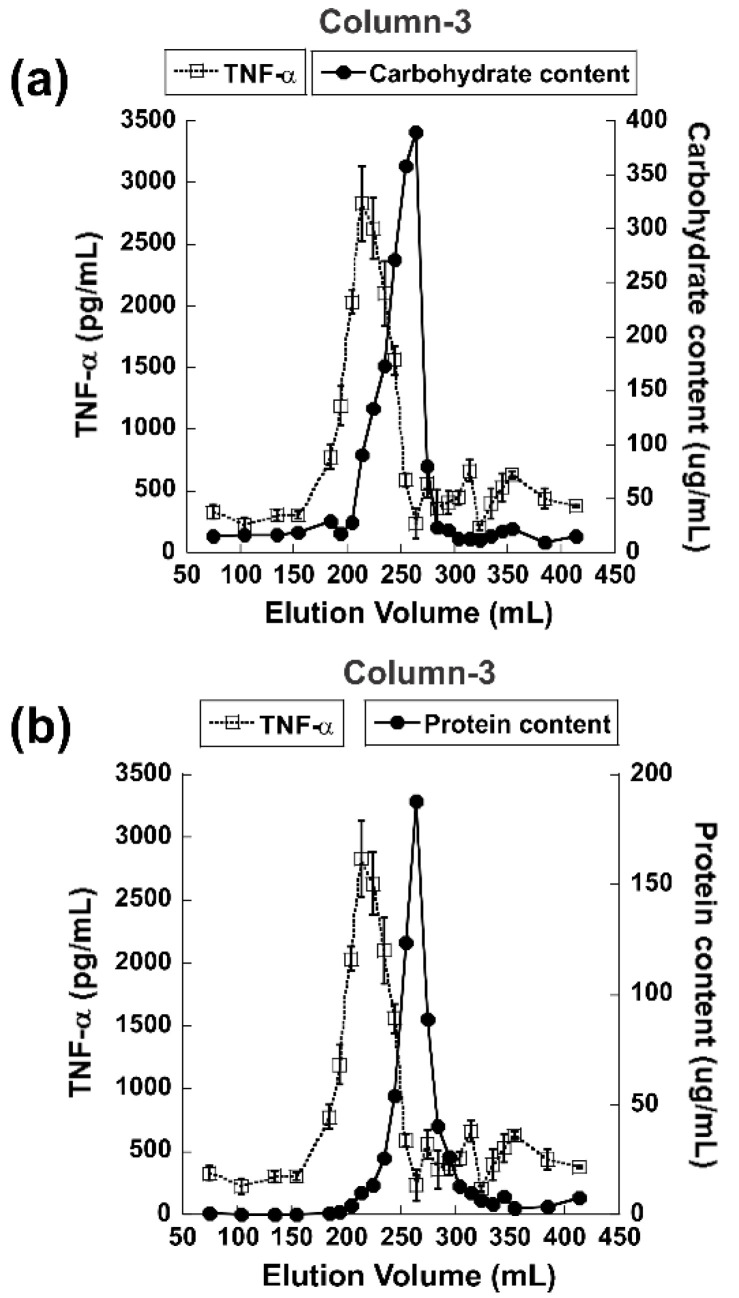
Purification of IMPP-Rb from *R. badius* using Sephacryl S-500 (column 3). Collected fractions were assessed for their ability to induce TNF-α production in RAW264.7 cells. TNF-α levels plotted against (**a**) carbohydrate contents, and (**b**) protein contents. Results are representative of three separate experiments.

Figure 2b shows the dose-dependent immunomodulatory activity of both the eluent and the flow-through obtained from a run in column 2. As shown, the eluent retained most of the activity, while no activity was observed in the flow-through. The dialyzed and lyophilized eluent 2A from column 2 was then subjected to further purification using column 3 (Sephacryl S-500 size-exclusion chromatography), and the results are shown in Figure 3. Figure 3a,b shows results from the Sephacryl S-500 run where the fractions were subjected to carbohydrate and protein content analyses, respectively, in addition to analysis for immunomodulatory activity. The immunomodulatory activity peak obtained from fractions 21–29 (elution volume 170–260 mL) was designated 3A. Interestingly, there was an overlap between the immunomodulatory activity and carbohydrate content, although there was no absolute correlation (Figure 3a). According to protein content analysis (Figure 3b), the peak shifted even more to the right, away from the peak of immunomodulatory activity, suggesting greater correlation of the bioactive compound(s) with carbohydrate than with protein content.

**Figure 4:**
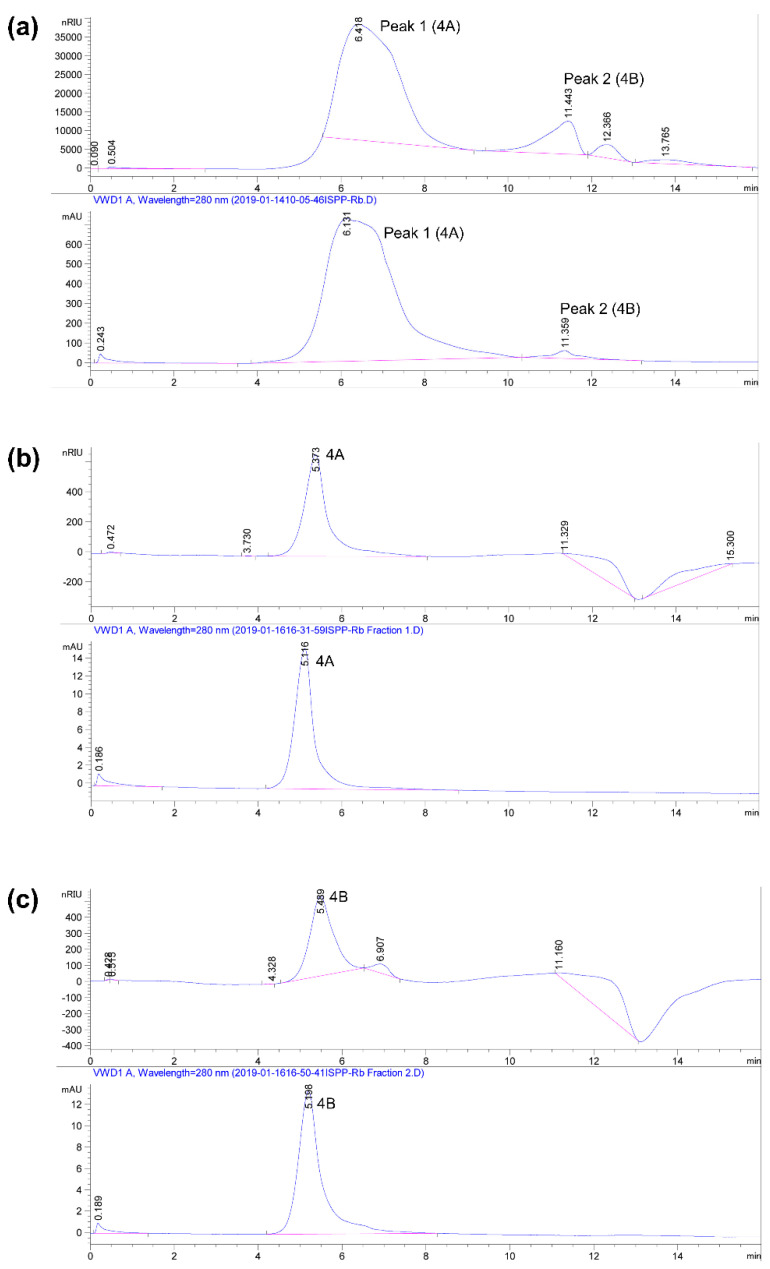
Purification of IMPP-Rb from *R. badius* using HPLC BioSEC5 (column 4). (**a**) Pooled, dialyzed, and lyophilized bioactive fractions from Sephacryl S-500 (column 3) were injected into BioSEC5. (**b**) Peak 1 (6–7.5 min) purified from (**a**) was reassessed for purity using BioSEC5. (**c**) Peak 2 (11–11.6 min) purified from (**a**) was reassessed for purity using BioSEC5. The top panel shows the refractive index spectrum and bottom panel indicates the UV spectrum at 280 nm.

In an attempt to purify the immunomodulatory compound to homogeneity, 3A fractions from several Sephacryl S-500 columns were pooled, dialyzed, and lyophilized for high-resolution HPLC BioSEC5 size-exclusion chromatography, before being subjected to column 4. Figure 4a shows the HPLC profiles of 3A. Peaks 1 (designated 4A) and 2 (designated 4B), with retention times of 6–7.5 min and 11–11.6 min, respectively, were collected separately and their purity re-assessed using column 4. Figure 4b shows that 4A was a relatively pure compound with a retention time of 5.373 min. Figure 4c shows that 4B was a major compound with a retention time of 5.489. Given its relatively pure state, the bioactive compound in 4A was selected for further chemical and biological analysis and was named immunomodulatory polysaccharide–protein from *R. badius* (IMPP-Rb).

To estimate the size of IMPP-Rb, T-series dextrans with molecular weight ranging from 150–2000 kDa were used on BioSEC5 (Figure S1). Based on the results, the peak maxima molecular weight (M_p_) of IMPP-Rb was estimated to be 950 kDa. We performed further calculations to determine the number-average (M_n_) and weight-average molecular weight (M_w_) of IMPP-Rb. As shown in Table S1, the M_n_ and M_w_ of IMPP-Rb were calculated as 949 kDa and 1001 kDa, respectively. The polydispersity index was calculated as 1.05. The carbohydrate content of IMPP-Rb, as determined by GC-FID, was estimated to be 57.4 ± 2.1% (% *w/w*), and its protein content, as determined by BCA assay, was estimated to be 16.7 ± 1.7% (% *w/w*).

### 3.4. Chemical Analysis of IMPP-Rb

**Figure 5:**
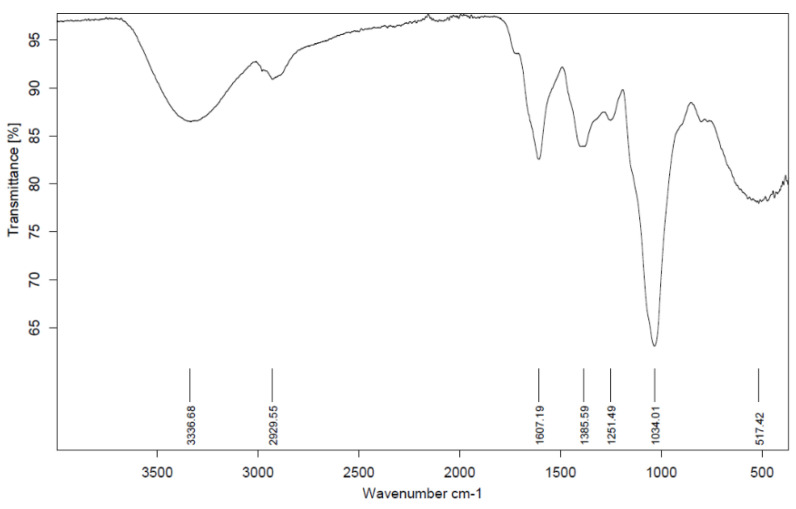
FTIR spectrum of IMPP-Rb.

IMPP-Rb was first subjected to functional group analysis using FTIR in the frequency range 4000–400 cm^−1^ (Figure 5). The stretches observed in IMPP-Rb are typical of fungal polysaccharides. The FTIR spectrum of IMPP-Rb showed a broad OH stretch at 3336 cm^−1^, sp^3^ C-H stretches at 2929 cm^−1^, and stretches at 1034 cm^−1^ for C-O for the pyranose ring. GC-MS analysis identified a band at 1607 cm^−1^ indicating a carbonyl C=O that might be due to galacturonic acid (Table 1 and Table 2). The bands at 1385 cm^−1^ and 1251 cm^−1^ indicate C-O-H stretching in carboxylic acids and C-O stretching in carboxylate and/or ether groups, respectively.

Monosaccharide analysis with TMS derivatization using GC-MS revealed that IMPP-Rb consisted predominantly of glucose (Glc) (49.2%), galactose (Gal) (11.3%), and mannose (10.8%) (Table 1, Figure S2). Relatively large amounts of rhamnose (Rha) (9.6%) and galacturonic acid (GalA) (8.2%) were also found. Several other glycosyl residues were also found in small amounts, including xylose (Xyl) (5.2%), fucose (Fuc) (2.8%), N-acetylglucosamine (GlcNAc) (1.8%), and arabinose (Ara) (1.2%) (Table 1).

Consistent with the composition analysis, the glycosyl linkage analysis showed multiple glucose linkages in IMPP-Rb (Figures S3 and S4 and Table 2). The main glucose linkages were 4-substituted, terminal, 3-substituted, and 3,6-, 4,6- and 3,4-disubstituted. Other glycosyl linkages present in relatively high amounts included4-substituted mannose, terminal mannose, 6-substituted mannose, 3,4-disubstituted mannose, 2,4-disubstituted galactose, terminal galactose, 2-substituted rhamnose, 4-substituted xylose, terminal fucose, and 4-substituted galacturonic acid.

### 3.5. Immunomodulatory Activity of IMPP-Rb

A mouse cytokine/chemokine 32-plex array was employed to determine the cytokine secretion profile of RAW264.7 cells after 6 h treatment with IMPP-Rb. The results are summarized in Table 3. IMPP-Rb treatment markedly enhanced the secretion of cytokines, ranging from an increase of 2.3 to 927 times higher than the control. The cytokines that were most significantly induced (>10-fold increase) included granulocyte-colony stimulating factor (G-CSF), granulocyte macrophage-colony stimulating factor (GM-CSF), interleukin-6 (IL-6), interleukin-17 (IL-17), interferon gamma-induced protein-10 (IP-10), leukemia inhibitory factor (LIF), macrophage inflammatory protein-2 (MIP-2), macrophage inflammatory protein-1α (MIP-1α), and macrophage inflammatory protein-1β (MIP-1β). Cytokines that were moderately induced (2- to 10-fold increase) included interleukin-1α (IL-1α), interleukin-1β (IL-1β), interleukin-2 (IL-2), interleukin-4 (IL-4), interleukin-10 (IL-10), interleukin-12 (IL-12), interleukin-15 (IL-15), and RANTES (regulated on activation, normal T cell expressed and secreted).

### 3.6. Effect of Proteinase-K on IMPP-Rb Immunomodulatory Activity

**Figure 6:**
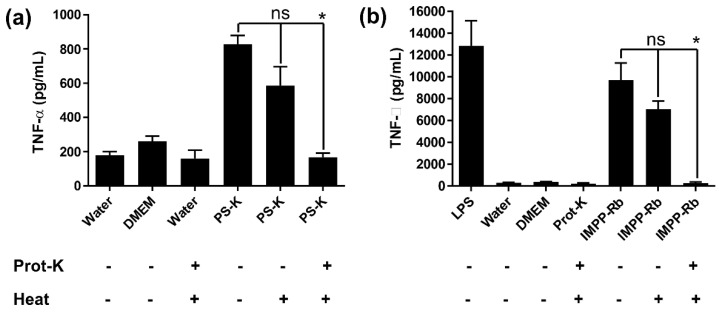
Effect of proteinase-K on immunomodulatory activity of polysaccharides. (**a**) PS-K (0.2 mg/mL) and (**b**) IMPP-Rb (1.5 mg/mL) were treated with 1 mg/mL proteinase-K. Samples were then heated at 80 °C for 20 min before being added to RAW264.7 cells. Results shown are mean ± SD from three independent experiments. One-way ANOVA (Tukey test) was used for statistical analysis. ns = not significant. * *p* < 0.05.

The results presented in Figure 3c suggested the presence of a protein component in IMPP-Rb, and this was further confirmed using the BCA assay. Therefore, it is of interest to determine whether the protein component in IMPP-Rb is important for its immunomodulatory function. PS-K is a polysaccharide–peptide complex and its peptide component is known to be critical for its immunomodulatory activity [25]. Therefore, PS-K was used as a positive control. As indicated in Figure 6a, pre-treatment with proteinase K completely abrogated the immunomodulatory activity of PS-K. Heating was included in the procedure to inactivate the proteinase-K which may otherwise have had an effect on RAW264.7 cells. The results shown in Figure 6b reveal that pre-treatment of IMPP-Rb with proteinase-K completely abrogated its immunomodulatory activity. Based on these results, it is concluded that, similar to PS-K, the protein component of IMPP-Rb is essential for its immunomodulatory activity.

## 4. Discussion

The potential medicinal properties of *Royoporus badius* have not been well studied. Previous reports have described the antibacterial [19] and anti-oxidant activities [20,21] of its ethanol and water extracts, respectively. In a separate study, zymosan-induced phagocytosis of isolated human neutrophils revealed that ethanol extract of *R. badius* had no significant effect on immunomodulatory activity *in vitro* [26]. Such observations appear consistent with our finding that ethanol extract from *R. badius* exhibited only modest immunomodulatory activity (Figure 1c,d). In addition, we report here for the first time strong immunomodulatory activity from water and methanol extracts of *R. badius*, and have taken the initiative to purify and characterize this potent immunomodulatory compound from *R. badius*.

The use of ion-exchange and size-exclusion chromatography, including HPLC, enabled the purification of IMPP-Rb from the water extract of *R. badius*. IMPP-Rb was confirmed to contain carbohydrate and protein components. FTIR analysis showed the presence of OH, C-H, C-O, and C-O-H stretches in the spectra, typical of fungal polysaccharides. A carbonyl C=O band at 1607 cm^−1^ was additionally observed, suggesting the presence of carboxylic groups in IMPP-Rb. This suggestion is further corroborated by the presence of galacturonic acid as determined by GC-MS. Present at almost 50%, glucose is the major monosaccharide in IMPP-Rb; other monosaccharides found in relatively high amounts include galactose, mannose, rhamnose, and galacturonic acid. The presence of high levels of 4-substituted glucose (20.7%) and 4-substituted mannose (7.7%) suggests that the major backbone in IMPP-Rb is a 1,4-glycosidic linkage; 1,3- and 1,6-glycosidic linkages are also likely to be prominent due to relatively high quantities of 3-substituted glucose (5.2%), 6-substituted mannose (4.2%), and 3-substituted mannose (3.5%). The presence of high amounts of di-substituted glucose, mannose, and galactose indicates that IMPP-Rb is a highly branched polysaccharide. IMPP-Rb includes relatively high amounts of galacturonic acid and rhamnose, further distinguishing it from other known fungal polysaccharides or fungal polysaccharide–protein complexes.

Based on the above information, we conclude that IMPP-Rb is a highly heterogeneous heteroglucan protein. Many heteroglucans have been isolated from fungi, but none have monosaccharide compositions, ratios, or types of linkages that are similar to those described for IMPP-Rb [7,27,28]. A few polysaccharide–protein complexes with the ability to stimulate immune cells have been isolated from mushrooms, but none exhibit molecular weight and composition resembling IMPP-Rb [7,27,29,30]. For instance, an immuno-stimulating proteoglycan with anti-cancer activity in vivo was isolated from *Trametes robiniophila*, but it is 55.9 kDa in size and has xylose as its predominant monosaccharide [30]. A water-soluble polysaccharide–protein complex with immunomodulatory activity was isolated from *Polyporus rhinocerus*, but it is 50 kDa in size with a high rate of 1,2-Man*p* linkage that is absent in IMPP-Rb [15]. Likewise, a 980 kDa immuno-stimulatory polysaccharide was recently purified from the wild mushroom *Lactarius deliciosus*, but its composition and linkages remain unknown because detailed chemical analysis was not performed [31].

The ELISA experiments confirmed the immunomodulatory effect of IMPP-Rb on murine macrophage cells in vitro. In addition to inducing TNF-α, IMPP-Rb could greatly enhance the secretion of G-CSF, GM-CSF, IL-6, IL-12, IL-17, IP-10, LIF, MIP-2, MIP-1α, MIP-1β, and RANTES. IL-6 is known to be a key cytokine for stimulating T- and B-cell differentiation and the activation of macrophages [32,33]. IMPP-Rb significantly induced M1 chemokines including RANTES, MIP-1α, and MIP-1β, as well as M2 chemokines such as MIP2, suggesting that it could induce both Th1 and Th2 immune responses [34].

However, it is important to note that our study cannot distinguish whether IMPP-Rb truly exerts an immunomodulatory effect leading to anti-cancer activity [7,35] and/or induces inflammation in animals. To determine this and to better understand the possible mechanistic effects exerted by IMPP-Rb, additional in vitro and animal studies will b necessary. Our study shows that, similar to PS-K, the protein component of IMPP-Rb is critical for its immunomodulatory activity. Future studies on IMPP-Rb should include detailed investigation into its mechanism of action and analysis of the protein component of IMPP-Rb, including determining its amino acid sequence and whether it is covalently linked to the carbohydrate component, or was simply co-purified with it. It would also be interesting to know whether the carbohydrate component is required to enable the immunomodulatory activity of IMPP-Rb.

## 5. Conclusions

This is the first study describing the isolation of a potent immunomodulatory compound from the polypore fungus *R. badius*. Using DEAE-Sephadex, Sephacryl S-500, and BioSEC5, we successfully isolated a 950 kDa immunomodulatory heteroglucan protein complex (IMPP-Rb) from the water extract of *R. badius*. IMPP-Rb is composed predominantly of glucose, galactose, mannose, rhamnose, and galacturonic acid, with smaller amounts of xylose, fucose, N-acetyl glucosamine, and arabinose. The protein component of IMPP-Rb is indispensable for its immunomodulatory activity. In addition to inducing TNF-α, IMPP-Rb was found to enhance significantly the levels of G-CSF, GM-CSF, IL-6, IL-17, IP-10, LIF, MIP-2, MIP-1α, and MIP-1β in mouse macrophage cells in vitro.

## Figures and Tables

**Table 1:** Monosaccharide composition of purified immunomodulatory polysaccharides from *R. badius* (IMPP-Rb).

Monosaccharide Residue	Mass (µg)	Mol %
Glucose (Glc)	12.7	49.2
Galactose (Gal)	2.9	11.3
Mannose (Man)	2.8	10.8
Rhamnose (Rha)	2.3	9.6
Galacturonic acid (GalA)	2.3	8.2
Xylose (Xyl)	1.1	5.2
Fucose (Fuc)	Trace	2.8
N-Acetyl glucosamine (GlcNAc)	Trace	1.8
Arabinose (Ara)	Trace	1.2
Total=		100

**Table 2:** Relative area values from glycosyl linkage by monosaccharide species in IMPP-Rb.

Monosaccharide	Linkage Type	Relative Area (%)
Rhamnose (Rha)	t-Rha	1.3
	2-Rha	3.1
Fucose (Fuc)	t-Fuc	2.1
Xylose (Xyl)	4-Xyl	3.5
Galacturonic acid (GalA)	t-GalA	1.2
	4-GalA	2.9
Mannose (Man)	t-Man	8.2
	3-Man	3.5
	4-Man	7.7
	6-Man	4.2
	3,4-Man	5.4
	4,6-Man	1.1
Galactose (Gal)	t-Gal	1.6
	3,4-Gal	1.2
	2,4-Gal	1.7
	4,6-Gal	1.1
Glucose (Glc)	t-Glc	8.0
	3-Glc	5.2
	4-Glc	20.7
	3,4-Glc	1.9
	3,6-Glc	3.1
	4,6-Glc	2.6
	3,4,6-Glc	1.1
	2,3,4,6-Glc *	1.9

* This fully substituted glucose residue was probably an artifact created by incomplete methylation. For clarity, residues or linkages found to be < or equal to 1% were omitted.

**Table 3:** Cytokine induction by IMPP-Rb in RAW264.7 murine macrophage cells.

Cytokine (Abbreviation)	Cytokine (Full Name)	Fold ± S.D. *
CCL11	Eotaxin	1.86 ± 1.21
G-CSF	Granulocyte-colony stimulating factor	927.18 ± 1122.83
GM-CSF	Granulocyte macrophage-colony stimulating factor	138.01 ± 158.85
IFNγ	Interferon gamma	1.00 ± 0
IL-1α	Interleukin-1α	3.93 ± 0.73
IL-1β	Interleukin-1β	2.34 ± 0.76
IL-2	Interleukin-2	3.38 ± 0.85
IL-4	Interleukin-4	2.56 ± 0.39
IL-5	Interleukin-5	1.00 ± 0
IL-6	Interleukin-6	22.32 ± 26.75
IL-9	Interleukin-9	1.15 ± 0.06
IL-10	Interleukin-10	4.22 ± 3.45
IL-12 p70	Interleukin-12	7.31 ± 6.75
IL-15	Interleukin-15	3.65 ± 2.02
IL-17	Interleukin-17	20.4 ± 0.42
IP-10	Interferon gamma-induced protein-10	463.94 ± 574.54
LIF	Leukemia inhibitory factor	121.06 ± 103.07
MCP-1	Monocyte chemo attractant protein-1	1.61 ± 0.32
MIP-2	Macrophage inflammatory protein-2	224.13 ± 19.63
MIP-1α	Macrophage inflammatory protein-1α	39.68 ± 29.28
MIP-1β	Macrophage inflammatory protein-1β	445.88 ± 113.55
RANTES (CCL5)	Regulated on activation, normal T cell expressed and secreted [Chemokine (C-C motif) ligand 5]	8.10 ± 3.37
TNF-α	Tumor necrosis factor-α	144 ± 43.14

* Fold changes are relative to the control (150 mM NaCl). The mean fold change ± standard deviation (S.D.) is for dataset *n* = 2.

## Data Availability

Not applicable.

## Supplementary Materials

The following supporting information can be downloaded at: https://www.mdpi.com/article/10.3390/jof9010087/s1. Table S1, Calculating the number (Mn) and weight average molecular weight (Mw) of IMPP-Rb. Figure S1, Estimating the peak maxima molecular weight (Mp) of IMPP-Rb using HPLC BioSEC-5. Dextran standards and ISPP-Rb were loaded onto BioSEC-3 at a flow rate of 0.4 mL/min. This was used to convert the retention time (Rt) to retention volume, and then to the Average Distribution Constant (Kav) as shown in Table S1. Closed circles represent the Dextran Standards used and open squares are all points of the interpolated molecular weight distribution of IMPP-Rb. Figure S2, The GC-MS chromatogram from glycosyl composition analysis using TMS derivatization. Figure S3, GC-MS chromatogram resulting from glycosyl linkage analysis of neutral residues. Glycosyl linkage analysis was performed by partially methylated alditol acetates (PMAAs). Figure S4, GC-MS chromatogram resulting from glycosyl linkage analysis of uronic acid residues. Glycosyl linkage analysis was performed by partially methylated alditol acetates (PMAAs).
