# Supplementary material for: An Immunomodulatory Polysaccharide–Protein Complex Isolated from the Polypore Fungus Royoporus badius
Source: J Fungi (Basel). 2023 Jan 6;9(1):87. doi: 10.3390/jof9010087 (PMC9864380; doi:10.3390/jof9010087)
Supplement: Supplementary file 1 [file jof-09-00087-s001.zip › J Fungi_Electronic Supplementary Information.pdf]

Journal of Fungi

MDPI

Article

An Immunomodulatory Polysaccharide-Protein Complex Isolated from the Polypore Fungus *Royoporus badius*

Bryan C. C. Lim [1], Mehreen Zeb [1], Wai Ming Li [1], John Z. Tang [2], Christian Heiss [2], Linda E. Tackaberry [3], Hugues B. Massicotte [3], Keith N. Egger [3], Kerry Reimer [1] and Chow H. Lee [1,*]

[1] Department of Chemistry and Biochemistry, Faculty of Science and Engineering, University of Northern British Columbia, Prince George, BC V2N 4Z9, Canada

[2] Complex Carbohydrate Research Center, University of Georgia, Athens, GA 30602, USA

[3] Department of Ecosystem Science and Management, Faculty of Environment, University of Northern British Columbia, Prince George, BC V2N 4Z9, Canada

* Correspondence: chow.lee@unbc.ca; Tel.: +1 250 960 5413

Electronic Supplementary Information

Contents

Figure S1. Estimating the peak maxima molecular weight (M_p) of IMPP-Rb using HPLC BioSEC5……….......................2

Figure S2. The GC-MS chromatograms from glycosyl composition analysis using TMS derivatization…………………2

Figure S3. GC-MS chromatogram resulting from glycosyl linkage analysis of neutral residues…………………………..3

Figure S4. GC-MS chromatogram resulting from glycosyl linkage analysis of uronic acid residues……………………...3

Table S1: Calculating the number (M_n) and weight average molecular weight (M_w) of IMPP-Rb………………………...4

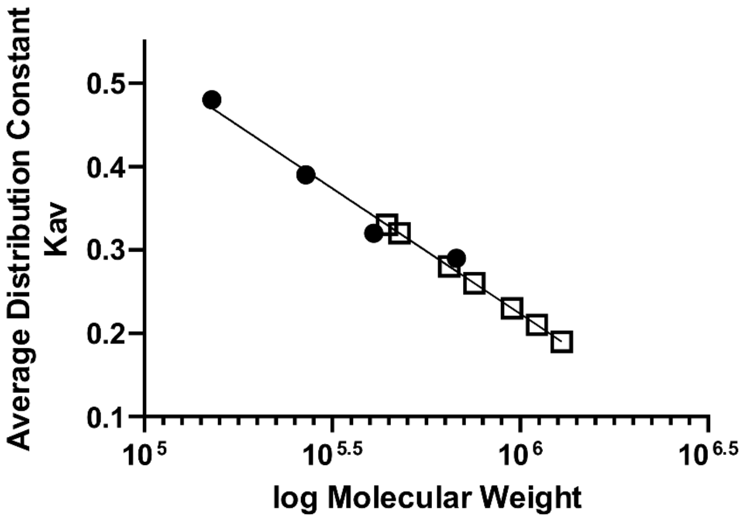

Figure S1. Estimating the peak maxima molecular weight (Mp) of IMPP-Rb using HPLC BioSEC-5. Dextran standards and ISPP-Rb were loaded onto BioSEC-3 at a flow rate of 0.4 mL/min. This was used to convert the retention time (Rt) to retention volume, and then to the Average Distribution Constant (Kav) as shown in Table S1. Closed circles represent the Dextran Standards used and open squares are all points of the interpolated molecular weight distribution of IMPP-Rb

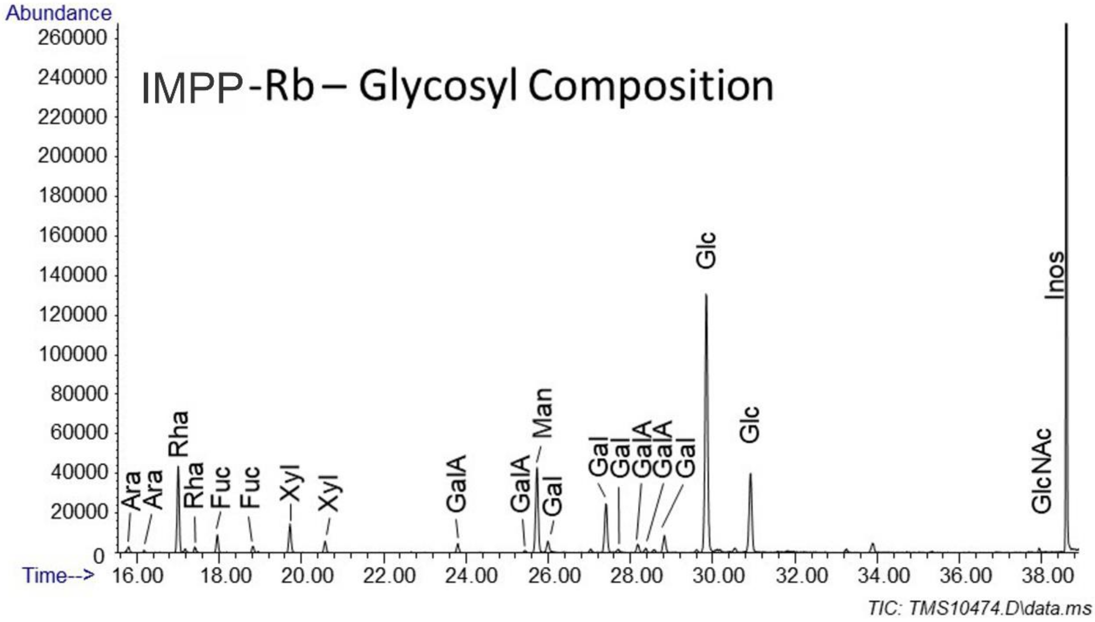

Figure S2. The GC-MS chromatogram from glycosyl composition analysis using TMS derivatization.

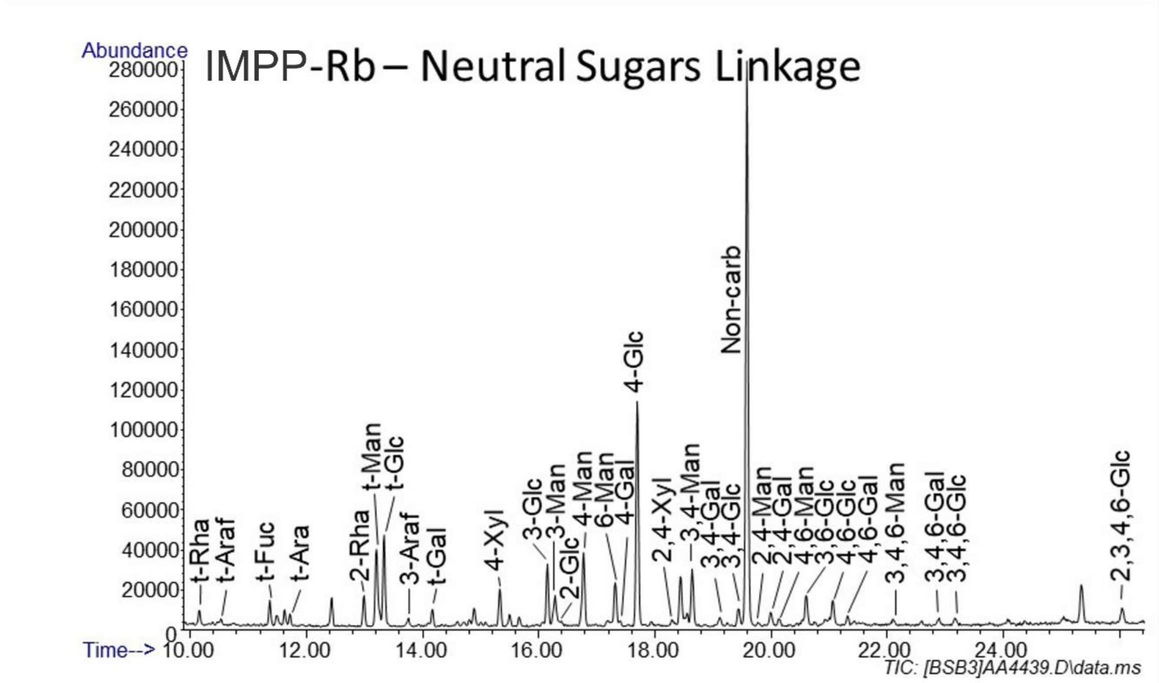

Figure S3. GC-MS chromatogram resulting from glycosyl linkage analysis of neutral residues. Glycosyl linkage analysis was performed by partially methylated alditol acetates (PMAAs).

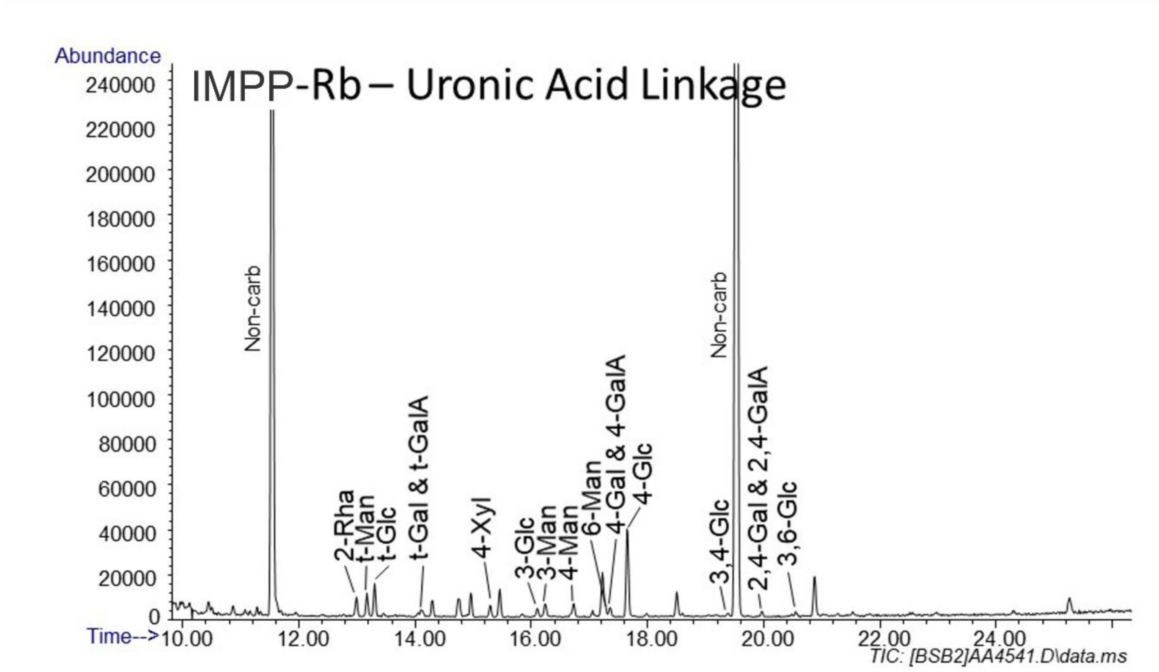

Figure S4. GC-MS chromatogram resulting from glycosyl linkage analysis of uronic acid residues. Glycosyl linkage analysis was performed by partially methylated alditol acetates (PMAAs).

Journal of Fungi

Table S1. Calculating the number (M_n) and weight average molecular weight (M_w) of IMPP-Rb.

Rt time (min)	Rt volume (mL)	Ve-Vo	Kav=Ve-Vo/Vc-V0	Mwt (log)	MW or Mi (g/mol)	Moles	ni (number of molecules)	niMi	wi	wiMi
5.9	2.4	0.64	0.198142415	6.111	1291219	1.54892E-11	9.32762E+12	1.2044E+19	0.238966822	308558.50
6.1	2.44	0.68	0.210526316	6.044	1106623	1.8073E-11	1.08836E+13	1.2044E+19	0.238966822	264446.18
6.3	2.52	0.76	0.235294118	5.978	950604[a]	2.10393E-11	1.26698E+13	1.2044E+19	0.238966822	227162.81
6.5	2.6	0.84	0.26006192	5.878	755092	2.64868E-11	1.08836E+13	8.21809E+18	0.163056376	123122.56
6.7	2.68	0.92	0.284829721	5.812	648634	3.0834E-11	9.32762E+12	6.05021E+18	0.120043157	77864.07
						∑=	5.30922E+13	5.04003E+19		1001154.13[b]

Twenty mg of IMPP-Rb was loaded onto BioSEC-5 at a flow rate of 0.4 mL/min. Vo is the void volume or Rt volume of Dextran Blue 2000 = 1.76 mL. Vc = 1 Column volume = 4.99 mL. Therefore, Vc-Vo = 3.23 mL.

[a]Molecular weight peak maxima (M_p) = 950604 g/mol.

[b]Weight average molecular weight (M_w) = 1001154.13 g/mol.

Number average molecular weight (M_n) = 949297.6 g/mol. ($M_n = \Sigma niMi/\Sigma ni$)

Polydispersity index = 1.0546 (PDI = M_w/M_n)
